# Exploring Canadian Public Safety Communicator Mental Health Help-Seeking Behaviors

**DOI:** 10.1177/10482911251326648

**Published:** 2025-03-19

**Authors:** Emily Howe, Stephen Czarnuch, Rosemary Ricciardelli, Nadine Leduc

**Affiliations:** 1 School of Maritime Studies, 98014 Memorial University of Newfoundland Fisheries and Marine Institute, St. John's, Newfoundland and Labrador, Canada; 2 Department of Electrical and Computer Engineering, 7512 Memorial University of Newfoundland, St. John's, Newfoundland and Labrador, Canada; 3 7512 Memorial University of Newfoundland, St. John's, Newfoundland and Labrador, Canada

**Keywords:** public safety communicators, 911 dispatchers, public safety personnel, mental health, help-seeking

## Abstract

Public safety communicators are the first line of support for members of the public-facing emergency situations. Consequently, communicators are exposed to potentially psychologically traumatic events (PPTE) which are associated with an increase in the prevalence of mental health concerns. For communicators, PPTE exposure and the subsequent negative mental health consequences are exacerbated by low engagement in mental health help-seeking behavior. We surveyed (n = 361) Canadian public safety communicators, asking “What do you think stops people from getting help for their mental health” to identify, contextualize, and provide considerations about contributors to the lack of mental health help-seeking among communicators. Emergent theme analysis reveals 7 factors that circumvent help-seeking: access barriers; self-denial; consequences of seeking help; lack of knowledge; personal feelings; stigma and culture; and support. Discovering hindrances to help-seeking identifies how factors contribute to communications employee wellness and supports the creation of effective interventions and policy implementations to support communicator mental health.

## Introduction

Public safety communicators (eg, 911 call-takers, and dispatchers) are the first public safety personnel (PSP) to receive information about emergencies, crises, or calls for service.^1^ Similar to other PSP (eg, police officers, firefighters, paramedics, correctional workers), communicators are exposed to potentially psychologically traumatic events (PPTEs) that can have an impact on their mental well-being. They often encounter in rapid succession critical incidents that require high-stakes decision-making.^2,3^ Too often, communicators do not receive the opportunity for emotional closure because they do not learn the outcomes of emergencies. In addition, communicators are regularly exposed to distressing and abusive calls.^2,3^ Researchers have identified that regular and repeated exposure to PPTE is associated with an increased risk of screening positively for a mental health disorder.^4^

Communicators play an essential role within the PSP ecosystem. They are the first point of contact in an emergency, interacting with various PSP groups and the public.^1^ The communicator role is demanding. It requires the capacity to manage critical incidents, often in rapid succession, which is essential for the efficient and appropriate allocation of resources.^1,2^ At present, there is a paucity of tailored mental health support options for communicators,^19^ despite the population's high prevalence of positive screenings for mental health disorders.^4^ In a national Canadian survey, 33.2 percent of communicators screened positive for major depressive disorder, 18.3 percent for post-traumatic stress disorder (PTSD), 18.0 percent for general anxiety disorder, and 7.2 percent for alcohol use disorders. Overall, 48.4 percent screened positive for any mental health disorder.^6^ In addition, communicators report a higher prevalence of mental health disorders compared to diagnostic rates in the general public.^6^ A 2022 survey found that 18 percent of Canadians aged 15 and over met diagnostic criteria for an anxiety, mood, or substance use disorder.^7^ One reason for the lack of tailored support for communicators is a dearth of knowledge concerning the factors that contribute to their mental health help-seeking behaviors and hindrances to help-seeking—a *lacuna* we respond to by conducting this study. In this study, we identify barriers to mental health help-seeking that emerge from the qualitative analysis of open-ended responses of Canadian communicators to an online survey about their mental health. Knowledge of these barriers is necessary in order to be effective in encouraging communicators to seek the mental health supports they need.

### Mental Health in the Canadian Public

According to the most recent data available, in 2018, 5.3 million Canadians, nearly 1 in 5, reported needing help for mental health-related challenges, yet nearly half did not receive adequate support.^8^ In fact, 1.2 million Canadians reported their needs were partially met and 1.1 million reported their needs were not met.^8^ Citizens whose needs were unmet reported barriers to care, including a lack of knowledge about how to access services, financial barriers, or the inability to make time to seek and receive help.^8^ Additional barriers include fear of repercussions in the workplace, including negative treatment from management, discrimination from peers, and job loss.^9–11^

### Mental Health in PSP

As previously indicated, there is a paucity of information specific to PSP communicators. However, information relevant to the mental health of PSP generally is likely to apply to communicators. While 90 percent of citizens experience a PPTE in their lifetimes,^17^ PSP, including communicators, are exposed to diverse PPTE at more frequent rates.^4^ To exemplify, over 90 percent of PSP reported exposure to sudden death, serious transportation accident, and/or physical assault, while 51.8 percent of the general public reports exposure to death of a close family member due to violence, accident or disaster and 53.1 percent report exposure to physical or sexual assault.^17^ Such high frequencies of exposures to various PPTE, present a concern given repeat exposures to PPTE can be linked to mental health disorders (eg, post-traumatic stress disorder, general anxiety disorder, and alcohol use disorder).^18^Communicators are not exempt from such repeat exposures to PPTEs although they may be less visible than their frontline colleagues who directly engage with the public.

Despite regular exposure to PPTE, PSP tend to rely primarily on informal support systems such as spouses, friends, and co-workers. They seek professional help from physicians, psychiatrists, and employee assistance programs (EAP) only as a last resort.^5^ In a survey of PSP, 74 percent reported they would rely first on an informal mental health support (eg, a spouse). Respondents were prompted separately about several kinds of professionals who might provide support (eg, physician, psychologist, psychiatrist). For each type of professional, between 45 and 64 percent of respondents said they would never use that professional or use them only as a last resort.^5^ Although the routine exposure of PSP to PPTE and their limited tendency to seek formal help are well established, there remains a dearth of knowledge concerning the factors that contribute to communicators' mental health help-seeking behaviors. This information is necessary to effectively encourage communicators requiring mental health supports, to avail of professional services.

In PSP populations, access aids facilitated by employers^12,13^ and improved mental health knowledge from workplace training^14^ have been shown to encourage help-seeking behaviors for mental health.^15^ Meanwhile, factors such as perceived stigma^16^ have been identified as a hindrance to help-seeking.

### Stigma and Barriers to Treatment Seeking in PSP Populations

Stigma—a theory framing interpretations of mental health—is tied to an attribute marking an individual.^20^ Stigma can discredit a person and is too often attached to mental health.^21^ Mental health stigma consists of perceptions that a person who seeks mental health help is weak, incapable, and/or a failure.^15^ It is difficult to maintain a strong persona in PSP spaces^15^ in the face of stigma that deters disclosure of mental health issues through help-seeking.^15,21^ Among PSP professions, stigma toward mental health varies. For example, correctional workers display less stigma than firefighters.^15^ Krakauer and colleagues found a relationship between the stigma tied to mental health among Canadian communicators and their mental health knowledge, with increased mental health knowledge was associated with reduced levels of stigma.^15^

In the public safety communications workplace, paramilitary structures,^12^ unique workplace cultures and inherent risks may exacerbate stigma.^22,23^ Factors such as depersonalization, emotional detachment in the workplace, fear of career setbacks, and personal feelings of failure or guilt related to negative outcomes all contribute to the prominence of mental health stigma among PSP.^23,24^ Fear of repercussions from employers may be exacerbated by the lack of protective legislation when employees do disclose mental health issues or seek help.^10^ As previously indicated, despite an elevated prevalence of mental health disorders among PSP,^6,16^ individuals in this population demonstrate a lower propensity to seek help, suggesting that barriers to help-seeking behavior, including stigma, remain.^18^ Given their role in countering stigma, mental health educational programs for PSP may positively inform help-seeking.^14^ A 2020 study found that PSP who had completed mental health training courses were more likely to seek mental health assistance, compared to than PSP who had not completed such courses.^14^ Mental health education, even when focused on supporting the mental health of others and not personal needs, also supports treatment seeking.^14^

Negative experiences with mental health care providers can undermine efficacy of such services and become a barrier to treatment seeking. For instance, PSP who sought mental health treatment reported negative treatment-related experiences dissuaded them from future or continued help-seeking.^25,26^ Fortunately, the individuals affected sought alternative sources of help, such as online therapy^25^ or informal supports.^26^

### Current Study

In the current study, we aim to investigate factors associated with public safety communicators' mental health help-seeking behaviors in Canada. We analyzed qualitative responses to the open-ended question, “What do you think stops people from getting help for their mental health?” We strive, by identifying barriers to communicators seeking and receiving help, to inform future efforts to provide for better support the mental health of these essential PSP.

## Methods

We conducted an online survey with 179 items organized in 19 sections, primarily focused on understanding the self-reported mental health of Canadian public safety communicators. The survey included demographic questions and replicated the mental health self-screenings conducted by Carleton et al. in 2018.^6^ Additionally, we included several open-ended questions exploring various aspects of mental health in communicators. Central to the current study was the open-ended question “What do you think stops people from getting help for their mental health?” The questionnaire was available in both English and French and was administered through the Qualtrics survey platform provided by Memorial University of Newfoundland. Participants were recruited initially through the Association of Public-Safety Communications Officials (APCO) Canada by email, which provided potential participants with an anonymous link to the online survey tool.

Before completing the questionnaire, participants were provided with study information and were asked for their voluntary consent to participate in the study, after reading about the study objectives and methods. Further consent was obtained for direct quotation in publication, and only those participants who gave such consent at the end of the survey have been quoted in our results. To be included, participants had to be employed as communicators for OnStar, police, fire, or ambulance services in Canada. Following initial recruitment, we encouraged snowball sampling (ie, potential participants forwarding the anonymous study link to other communicators who were not members of APCO Canada) and contacted publicly available organizations (eg, provincial oversight organizations) to increase our sample. The Newfoundland and Labrador Human Research Ethics Board provided ethical clearances for our study (#20210168), and the survey was online from November 4, 2020, until April 30, 2021.

### Participants

A total of 361 participants responded to the prompt “What do you think stops people from getting help for their mental health?” Of our participants, 78.4 percent identified as women and 21.6 percent as men or other. Participants ranged from 22 years of age to 67. A plurality fall within the range of 30-39 years of age. Our communicators ranged from less than 1 year of experience to more than 10 years in their current roles. The plurality of participants had worked for more than 10 years in their current role. Roles held by our respondents include dispatcher, call-taker, trainer, manager/commander, director/chief, quality assurance/quality improvement (QA/QI), and IT/radio technician. Although some in our sample reported holding more than 1 current role, we reported their primary role only. The age, gender, occupational role, and participant number (represented as “P#”) are included as identifiers to accompany participant quotes in our results section. A summary of participant demographics can be found in Table 1.

**Table 1.** Summary of Participant Demographics (n = 361), With Response Categories Containing Fewer Than n = 5 Participants Combined With Smallest Category to Protect Anonymity.

Category	Sub-Category	Frequency (N)	Percent (%)
**Gender**	Man/other	78	21.6%
	Woman	283	78.4%
**Age**	20-29	55	15.2%
	30-39	126	34.9%
	40-49	108	29.9%
	50-59	58	16.1%
	60-69 or not indicated	14	3.9%
**Years in role**	Less than 1 year/Not indicated	34	9.4%
	1-2 years	45	12.5%
	2-5 years	80	22.2%
	5-10 years	85	23.5%
	10 + years	117	32.4%
**Role**	Call taker	70	19.4%
	Dispatcher	125	34.6%
	Director/Chief/Manager/Commander/Supervisor	63	17.4%
	Trainer	13	3.6%
	QA/QI	9	2.5%
	Other or multiple	81	22.4%

### Analyses

Survey data were analyzed with the support of QSR NVivo software. NVivo is a computer-assisted qualitative analysis software which allows for qualitative data management, analysis, and visualization by facilitating coding of transcribed participant responses into themes and subthemes.^27^ We developed a comprehensive codebook with mutually exclusive but exhaustive codes. Two researchers each independently coded responses to each question. Once agreement was achieved regarding the codebook, the researchers categorized responses under the appropriate thematic code (or node, as per NVivo). As more responses were reviewed, each coder was able to add new codes, with discussion, as themes emerged, amalgamate, or disaggregate codes. We also reclassified nodes, as per axial coding, a method which facilitates the breaking down of themes within a dataset by relating themes to each other, when nuance emerged that affected how words were operationalized.^28^ Researchers resolved any disagreements in coding with discussion.

## Results

**Figure 1.**
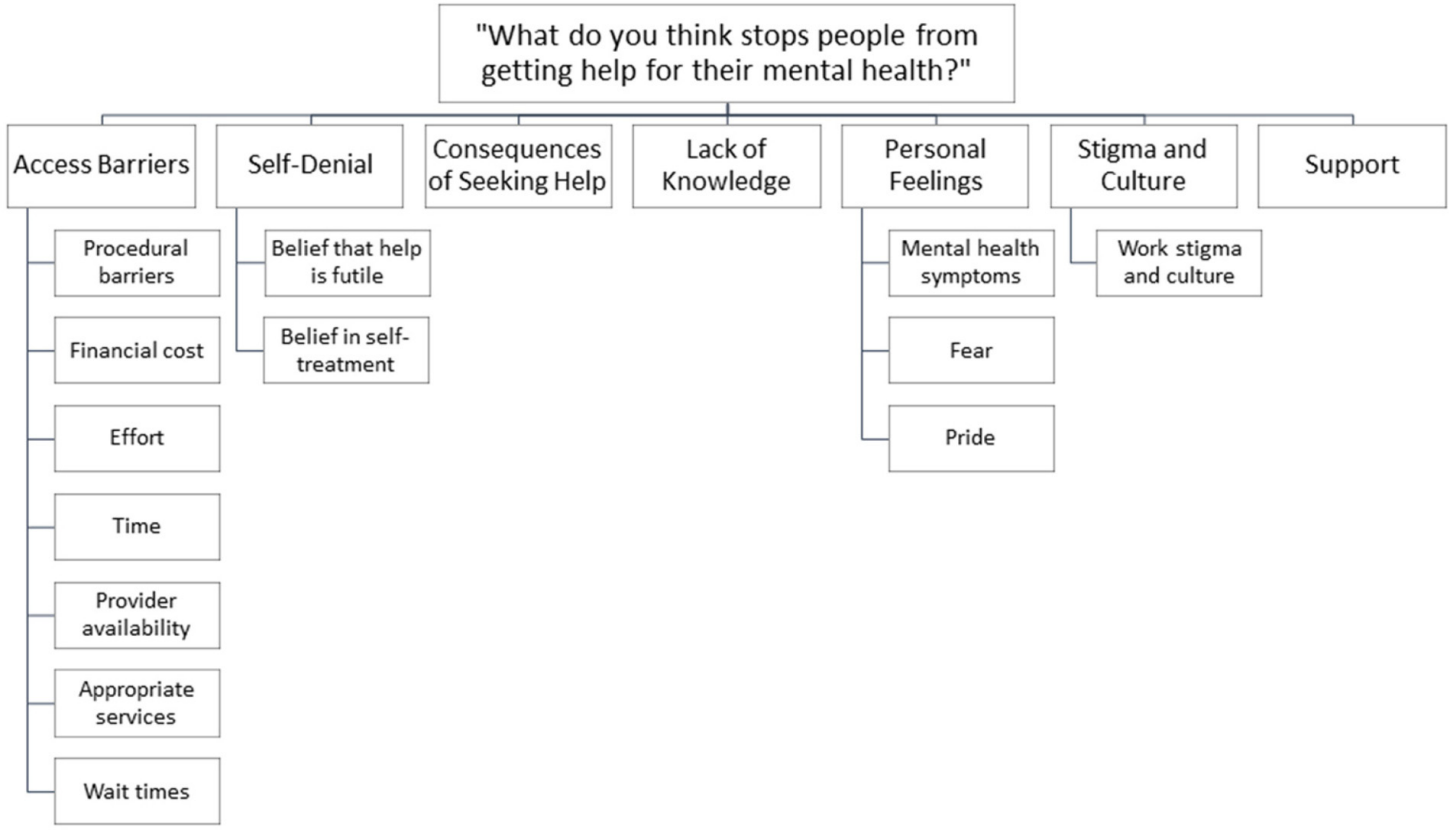
Major themes and sub-themes emerging from our analyses.

We unpacked the different barriers toward help-seeking our participants reported, identifying a total of 7 themes, with some sub-themes, each representing a barrier to mental health help seeking. The themes were: access barriers; self-denial; consequences of seeking help; lack of knowledge; personal feelings; stigma and culture; and support (see Figure 1). In general, an unwillingness to seek help emerged across respondents in our data. P91, for example, expressed “being a dispatcher/call taker we are always in control, asking for help means we have ‘lost control’. What makes us great at our jobs, makes us horrible at asking for help” (39, woman, call-taker/dispatcher). Reluctance to access and receive mental health help due to cultural norms and other factors within the workplace inhibited communicators from taking the first steps to access assistance.

*Access barriers*. Respondents described barriers to help-seeking. Many agreed with P411who stated that one barrier was “lack of access to proper mental healthcare” (27, man, dispatcher). We have categorized access barriers into the 7 sub-themes, to which we now turn.

*Procedural barriers*. Our participants expressed difficulty connecting with mental health supports due to administrative barriers, such as “red tape and runaround when it comes to resources” (37, woman, call-taker, P365). Others agreed that the internal organizational processes and external healthcare systems were challenging (eg, “Access. Especially in places where people have a hard time seeing their [family doctor] to get a referral” (37, woman, call-taker/dispatcher, P88)). Such thresholds for gaining access were viewed as a barrier to access of mental health support, creating a perceived “runaround” where one could easily feel trapped within systems rather than supported. The difficulty in accessing assistance is further compounded when “you already feel defeated and exhausted” (40, woman, supervisor, P303). P341 reports “when you are just trying to cope and get thru the day-to-day making extra appointments, calls, filling paperwork might be overwhelming” (34, woman, dispatcher, P341). The procedures for obtaining access can seem insurmountable, frequently stemming from bureaucratic complexities, creating an obstacle for individuals in need of treatment.

*Financial cost*. Our participants noted “mental health help costs money” (37, woman, call-taker/dispatcher, P88), and the coverage provided by benefits may be insufficient: “SHITTY BENEFITS; WE HAVE NO *REAL* SUPPORT AND CURRENTLY CAN GET 4 OR 5 SESSIONS BEFORE MAXING OUT…DAMN EXPENSIVE FOR SESSIONS 6+++” (49, man, dispatcher, P70, emphasis original). Many reported they were “not able to afford therapy” (38, woman, supervisor, P182) or wary of the potential “financial repercussions” (51, woman, manager/commander, P170) of mental health treatment.

Participants, such as P531, explained access is impeded by “the lack of coverage our benefits provide. With the minimal amount of coverage, people can get help (speak to a professional), maybe 3 times a year” (32, woman, call-taker/dispatcher). A considerable number of participants believed that their employers should assume responsibility for the expenses associated with support services: “Access to professional mental health care workers and lack of funds to pay for it. Even if its reimbursed, it should be paid for by the police agency I work for directly” (40, woman, call-taker, P180). Insufficient benefits posed a significant barrier, as highlighted by P20, “Knowing what little coverage, I have stops me from even going to the one or two sessions that my health benefits would cover, because I know it's not enough and will just frustrate me further” (40, woman, trainer).

*Effort.* The actual or anticipated effort required to receive mental health help deterred some participants from seeking treatment. Communicators felt they lacked the required “energy” (41, woman, call-taker/dispatcher, P64) to access services. The discouraging effect of having little energy due to the fatigue from communicators work, interacted with effort requirements to obtain help. Here, P325 wrote “we're just so exhausted after a work block that we don't want to leave the house or talk to anyone. Just feel that we don't have the energy … emotionally exhausted” (44, man, manager/commander).

*Time.* The time required to attend to their mental well-being was also a deterrent, as “people don't want that time commitment” (44, man, manager/commander, P325). Restrictions on communicators' time, particularly allowances for time off work, further affected help-seeking. For example, according to P148 there was “NO AVAILABLE PAID TIME OFF FOR MENTAL HEALTH” (34, woman, dispatcher, emphasis original). Moreover, communicators who worked full-time hours but were not designated full-time employees found it particularly frustrating that they were unable to take time off due to financial constraints. P258, to exemplify, wrote “As a casual employee who works full time hours (my work place will not create more full time positions and abuses the term “casual”) I cannot afford time off work to seek out medical help for mental health issues” (30, woman, call-taker/dispatcher).

*Provider availability.* Participants felt confined by little “access to professional mental health care workers” (40, woman, supervisor, P180) which affected their ability to seek help. For example, P77, a 57-year-old woman working as a call-taker expressed “[they] don't have enough people to see in our city” for psychological support. The absence of care providers was compounded by the fact that “psychologists, counsellors, nurses and doctors are too overworked and understaffed” (48, woman, call-taker/dispatcher, P394). Here, access to mental health care diminishes with human resource shortages, reducing available appointments with providers.

*Appropriate services.* Respondents noted feeling frustrated by a real or perceived “lack of access to proper mental health care” (27, man, dispatcher, P411). P225 reflected it was “hard to get appropriate treatment—and the trial and error is frustrating and detrimental” (49, woman, call-taker). The “trial and error” (P225) describes how appropriately different services can meet the mental health needs of communicators and affirms how the process of searching, and trying, for “appropriate treatment” can deter (even cease) mental health help-seeking.

Some participants in our sample did not find suitable treatment options through the use of services such as EAP. In the event that a communicator requires a more extensive treatment option than what is currently available through an EAP, they would need to seek out alternative treatment options by themselves, which could create a potential financial burden if coverage does not extend to the new provider. P317 wrote that “EAP only helps so much but there are people (such as myself) who need stronger, more serious treatment and it is difficult to access and afford” (31, woman, call-taker).

This lack of resources and treatments were possibly exacerbated by the COVID-19 pandemic, as some, like P355, experienced: “lack of follow through from health care because there are not enough resources available for everyone—especially now during the pandemic” (31, woman, dispatcher). When communicators are not provided with adequate and appropriate resources and treatments, including during times of increased need like the pandemic, their access to mental health care is limited, thereby potentially decreasing the probability of seeking or receiving assistance.

*Wait times.* Long wait times associated with receiving help were identified as access barriers by our participants. Respondents reported that “the wait times to see a professional are sometimes years long” (37, woman, advisor, P415), created by the high demand for mental health services, supported by P70 noted that “DUE TO THE O-V-E-R-W-H-E-L-M-I-N-G NEED EVERYWHERE THE DELAY TO ACTUALLY SIT WITH SOMEONE WHEN YOU DECIDE YOU NEED HELP IS EXCESSIVE” (49, man, dispatcher, emphasis original). The wait times reduced their desire for and openness to treatment and were such a deterrent, some respondents experienced fear or/and anxiety. For example, P50 wrote: “The fear of not getting to speak to someone right away when reaching out. The waiting period will deter someone” (40, woman, dispatcher). Substantiated by other respondents, they confirm experiencing long waiting periods which delay mental health help access or prevent future help-seeking.

*Self-denial*. Self-denial, referring to an inability to recognize personal mental health needs, or the denial of needing assistance when the mental health need is known, emerged among participants. Respondents, here, described an “inability to recognize there's an issue” (50, woman, dispatcher, P326) where one is not “accessing themselves and realizing they might now be in the category of people that need help” (26, man, call-taker, P293). This lack of self-awareness included “not making health a priority” (37, man, dispatcher, P60). Symptoms were thought to be unrecognized because of the collective nature of peers where many exhibit mental health challenges (eg, “failure to realistically recognize the severity of symptoms - in contrast to peers, are feeling ‘normal’ however peers are part of a micro group” (44, woman, dispatcher, P406)).

For others, denial attitudes prevailed: “people deny the signals for mental health issues” (32, man, dispatcher, P129) where people have difficulty “admitting to themselves they need help” (42, man, dispatcher P350). Denial also occurred when respondents admitted to having needs but felt they were “not that bad that I need help” (42, woman, trainer, P282). This minimization led respondents, like P146, to reflect on the feeling of not needing external help: “avoidance or they don't believe they need help or don't think they have a mental health problem” (33, woman, call-taker/dispatcher, P146). The theme of denial yielded 2 major sub-themes: the belief one's mental health cannot be helped, and the feeling one can manage their mental without help.

Regarding the former, the belief help is futile, respondents detailed how someone may recognize their need but believe treatment is not worth seeking because they cannot be helped. P412 wrote “sometimes it's just admitting you need help and the willingness to seek it out. But most times it seems to be that sense of ‘what's the use, nobody can help me’” (48, woman, call-taker). The perception of mental health as untreatable was made in contrast to physical injury by some respondents, such as P8, who reported feeling that, unlike a physical injury, their mental health will never heal. They said: “People feel that once you have it you always have it to the same level, and that it never really goes away. Unlike a broken leg you can repair and often times like new or better” (47, woman, quality assurance/quality improvement (QA/QI)). A “feeling of hopelessness, and that maybe nothing will help” (41, woman, dispatcher, P637) also contributed to a disregard for help-seeking, which some thought was useless because they felt unresponsive to treatment.

Regarding the latter, belief in self-treatment, participants felt personally managing their mental health needs was a viable option. Respondents reported “thinking they're ok, that they don't need help, and they can just work through it” (43, woman, dispatcher, P308). Believing that one can be self-sufficient in addressing mental health, can lead to denial of the value of external mental health supports, and, for that reason, declining to seek access. Respondents combined this sentiment with the tendency to “downplay things” thinking “well my problems aren't that bad so I should really just learn to deal with it” (31, woman, dispatcher, P62) to inhibit treatment seeking. Participants also attributed learning to feel they should be self-sufficient to their work environment. For instance, P185, a 49-year-old woman working as a dispatcher said:

For someone like a communications officer who has received more than the average training on coping with stress it becomes extremely hard to admit that you can no longer cope or are struggling. You feel that you should know better with all the education received to cope.

*Consequences of seeking help*. Respondents wrote of apprehension about what might happen after initiation of help or due to past experiences with mental health intervention as a barrier. P190 shared the negative and harmful aspects of their prior mental health seeking attempt:

The mental health care/system is terrible. The “help” is not always beneficial it sometimes it sets the person further back than helping them get a head. I myself experienced a stay at the hospital and the psychiatrist who was evaluating me was manipulative and asked me “do you think you're better than everyone on this floor? If I was still in the fragile state I was when I first came in it probably would have pushed me to attempt suicide. (27, woman, call-taker)

Negative interactions with providers were echoed by other respondents, like P188, who wrote people are “NOT TRUSTING THE PROFESSIONALS THEY MEET” as they feel “THEY WON'T UNDERSTAND” (42, man, call-taker, emphasis original). The barrier of health care providers not “understanding” was frequent, with P74 describing how a “Lack of understanding in the health care profession for the stressors and secondary stress indicators that are regularly seen and dealt with in our field” (40, man, supervisor).

Apprehension about the opinion of and reaction from others when seeking treatment, such as family, friends, and colleagues, was also worrying for respondents. Many expressed “the worry of people finding out that they are seeking help for a mental health concern” because doing so “can still be widely frowned upon” (45, woman, call-taker, P379). Here is evidence that mental health is stigmatized: “The idea, real or imagined, that others will find out and judge you for having mental health problems” (33, woman, dispatcher, P268). Respondents also expressed being “worried about being labelled by peers” (26, woman, call-taker/dispatcher, P184) should their mental health challenges become public knowledge in the workplace. P640 worried they would be “labelled and socially outcast” (43, man, supervisor). These quotes indicate that stigma continues to frame mental health. Stigma included fear that superiors would adopt of negative opinions of those whose mental health challenges became known. Respondents indicated “They don't want to be seen by management as ‘weak’” (58, woman, dispatcher, P219). Details of a “Lack of genuine support from management” (43, woman, call-taker/dispatcher, P25), where “time constraints” (33, woman, dispatcher, P252) because respondents did not believe management was understanding and, in response, would hinder their ability to obtain required time off work for mental health care.

In addition, fear for “implications to their employment” (P381) included concerns of losing opportunities for career advancement by “being passed over for promotions” (47, woman, call-taker, P381) should the employer learn about their mental health challenges. Others feared job loss: “it opens a door to being fired or forced to quit” (46, woman, call-taker/dispatcher, P157). Worries around loss of income also arose. Should an employee's mental health challenges be made known in the workplace, respondents such as P277 indicated supervisors “perceive that they will be unfit for the job requirements and not be able to do the job anymore. This is led by fear of significant loss of potential future income” (42, man, dispatcher). Thus fear of repercussions from colleagues and management for seeking mental health support remains a barrier.

*Lack of knowledge*. Some communicators described a “lack of knowledge” (42, woman, dispatcher, P242) and “not knowing what their options are” (42, woman, supervisor, P399) for available mental health resources or how to access them. Regarding the former, participants described a lack of education on resources and treatments. They detailed “misunderstanding of treatments” (36, man, call-taker/dispatcher, P17) and not knowing “what to expect of that help—meds, doctors, feedback from family/work” (45, woman, dispatcher, P409) leading to apprehension, which constitutes a barrier to mental health help-seeking. Limited awareness on how to initiate access to mental health help was pronounced among respondents. As P317, a 31-year-old woman working as a call-taker expressed “people also don't know where to get the right help” while others recounted “confusion on how to access services/the right ones” (25, woman, dispatcher, P56). Consistently here, respondents indicated “not knowing where to get help” (34, woman, call-taker/dispatcher, P28), deterring them from help-seeking.

*Personal feelings*. Personal feelings toward mental health affected help-seeking behaviors in 3 prevailing ways: mental health symptoms, fears, and pride, each of which we now unpack.

*Mental health symptoms*. Individual mental health status, for many respondents, confounded treatment seeking. Participants described “Depression, feeling lost” (age and gender not indicated, supervisor, P128) and “anxiety” (44, woman, call-taker/dispatcher/supervisor, P275) or even “Feeling unworthy of help” (37, woman, call-taker, P365) or “feeling alone in the issue” (32, woman, call-taker/dispatcher, P271). Symptoms experienced as part of pre-existing mental health conditions or needs appeared to impede treatment-seeking, with fear, pride, or embarrassment most commonly identified.

*Fear*. Beyond aforementioned fears, some respondents also indicated people have “fear of the process” of asking for help (33, man, dispatcher, P358). A “fear of change or what they might hear about their mental health” (32, man, call-taker/dispatcher, P24) emerged, with respondents describing fear towards confronting their mental health, which prevents help-seeking.

Some described fear in the realization that their mental health progressed into a severe state that was no longer manageable. P191, a 29-year-old woman working as a dispatcher wrote: “it's scary to admit that your coping mechanisms are no longer working.” Some respondents described being apprehensive about personal repercussions due to seeking mental health help with participants indicating fears of consequence towards their families, their finances, or towards their careers. This “concern that if they admit to having a mental health issue there may be other repercussions, (involvement of family services)” (35, woman, call-taker, P153) prevented people from admitting to and taking actions based on their own needs. This is compounded by a “fear of career consequences” (47, woman, business analyst, P343). Given these diverse fears, respondents such as P247 indicated they would prefer to suffer from poor mental health than seek assistance: “the one time I took short term leave was the highest my stress had been during my mental health issues. I'd rather suffer my mental health than have to deal with that additional stress to resolve it” (29, woman, call-taker/dispatcher).

*Pride*. Regarding pride, and accompanying embarrassment potential, participants reflected on how their “pride” (40, woman, call-taker/dispatcher/trainer, P173) contributed to being “embarrassed to share how they are feeling” as they “think no one else will understand” (40, woman, call-taker, P256). Such pride was attributed to the “stigma in emergency services. If it bothers you, you're weak” (40, woman, call-taker/dispatcher/trainer, P173) or simply being “ashamed to ask for help” (34, woman, call-taker/dispatcher, P28).

*Stigma and culture*. “Stigma” (29, man, dispatcher, P205) and occupational culture were also put forth as deterrents to treatment seeking. Some respondents were “not wanting to be associated with the stigma of mental illness” (25, man, dispatcher, P206) due to “the pressures of society in general. Seeking mental health is still frowned upon” (32, woman, dispatcher, P299). Stigma around mental health also included association “with a weakness of character or inability to do your work” (47, woman, supervisor, P171) and being viewed by others “as crazy, unstable, unable to cope” (40, man, dispatcher, P276).

*Work stigma and culture*. Some respondents believed mental health stigma was intrinsic to workplace culture, which was expressed through comparing mental health stigma in communicator environments within society more broadly. For some respondents, workplace stigma is, at least in part, rooted in “workplace bullies talking about it. The employee doesn't want to be the next target” (46, man, manager/commander, P5).

Communicators describe, in their workplaces, “mental health is not a priority but mostly ridiculed. Rumors and the negative effects of being in a ‘family’ type organization discourages many people from seeking help” (44, woman, call-taker, P72, translated from French). Stigma around mental health was also ascribed to management. For example, P360, a 49-year-old woman call-taker, wrote: “The culture is such that talking about mental illness shows you are weak and unable to handle the job ‘you signed up for’ (a quote I have personally seen made by a manager to an employee in an email).” Thus, communicators culture perpetuated “the idea that communicators need to ‘suck it up’” (48, woman, dispatcher, P126).

As such, participants described feeling if they admitted to having mental health needs and sought assistance they would be viewed as unable to complete the occupational responsibilities. P68, a 31-year-old man working as a dispatcher detailed:

There is a stigma, particularly working as a 911 call taker/police call taker/dispatcher that having mental health issues would prevent you from being able to handle the job. Most of our interactions with the public are stressful, and regularly greatly upsetting in nature or violent, and there is a concern that people with [mental health and addiction] issues would not have the resilience to be able to withstand exposure to this without it having a negative impact on their mental health.

Respondents also noted their occupational training taught them to think and act stoically, to be calm under pressure, and to contain their emotions. “Adrenalin reactions, physical reactions etc. because otherwise we couldn't cope to get our job done. We are literally taught to keep going with whatever happens and try and ignore emotions” (35, woman, dispatcher, P95). Thus, the position teaches stoicism and suppression of emotions, which may impact coping processes and treatment seeking. P95, echoing others, felt that “no one is going to seek out help unless things get very bad” (35, woman, dispatcher). Compounding here are sentiments of having “signed up for it” (36, woman, dispatcher, P19) and being “viewed as being able to handle the trauma” (P19)—further deterring help-seeking. In consequence, respondents felt pressure to “power through” (32, woman, trainer, P216) their mental health challenges in light of workplace culture.

*Support*. Participants voiced concerns surrounding the quality of available support. Some, like P9, a 25-year-old woman working as a call-taker reported a “lack of personal/adapted help” while others wrote of a “lack of family support” (30, woman, trainer, P375) for mental health or a “lack of workplace supports” (45, man, supervisor, P140) where “employees are made to feel they aren't worth the help” (37, man, dispatcher, P253). Work support programs such as peer support and Workplace Safety and Insurance Boards (WSIB) were deemed inadequate by P140 who wrote “peer support is made up of managers not peers.” P140 also expressed a “misunderstanding that to access WSIB that you must be off work” (45, man, supervisor/manager). Thus, challenges with interpretations of how to use support alongside feeling unsupported prevailed.

## Discussion

Canadian public safety communicators may report higher service use intention compared to other PSP,^15^ but they still tend not to seek mental health support. In the current article, we interrogate why participants felt access to treatment, support, and intervention were laced with barriers to mental health help-seeking. These included emotional, social, psychological, and financial barriers (ie, money, effort, and time), all compounded by occupational factors tied to working within public safety. Moreover, even if these barriers did not exist, our respondents noted providers were hard to find or had long wait times, particularly those with appropriate training for communicator-related challenges.

Access barriers included denying the need for help, believing help is futile, or that self-management and self-treatment were sufficient. Reasons for not seeking help included feeling that seeking further treatment would be futile as well as denial or minimization of symptoms of mental distress. Communicators, like many people, may not know when help seeking is necessary. These findings are unsurprising: symptoms creep in intensity, leaving people unaware of the change in their personal wellness. Accordingly, providing communicators with more knowledge about how to recognize mental health deterioration and when help is needed, through mental health awareness training (see: Mental Health Commission of Canada;^29^ CIPSRT^30^) may encourage help-seeking behaviors as shown in other PSP populations.^14^ These findings support a potential positive correlation between recognition of a mental health need and increased likelihood of seeking help, clearly an area for further investigation among communicators to determine, for example (i) whether programming helps, (ii) types of helpful programming, (iii) how to deliver the programming, and (iv) the target audiences. Evidence suggests that programming and education could support help-seeking, given research with police officers shows education can be protective and encourage help-seeking.^12^

Communicators perceived help-seeking could result in adverse outcomes, which factored into the decision to seek care. Our participants fear that adverse outcomes would make their situation and health worse. This fear was attributable largely to a mistrust in the help providers, peers, or their employer/management, based on either direct personal experiences or a general belief. Participants articulated the belief that a communicator with a mental health disorder would be passed over for promotional opportunities or face workplace repercussions. Concerns about consequences arise from a workplace culture characterized by stigma, with a prevailing belief that communicators would be unable to perform their duties if they were struggling with mental health. They might be deemed unreliable in the workplace and weak. This finding is parallel to findings from other PSP.^16,22^

Some also felt knowing their mental health status was a disadvantage. This belief is indicative of self-stigma. It may also explain how fear and pride emerge as barriers. Relatedly, help-seeking was hindered by participants' worry about being judged or treated differently by family, friends, colleagues, or management. We found that perceptions of limited support from family and friends could bar help-seeking in some cases. This finding is consistent with research on broader PSP populations which has found that family and friend-based support is key to fostering mental well-being.^12,31–33^ Our finding that one barrier to help-seeking was the perception that supervisors were unsupportive of people with mental health needs is consistent with research on other PSP populations.^12^ Admitting to needing help could also at times be incompatible with one's self-concept, again due to stigma. Hence, communicators may be cyclically trapped. Their mental health symptoms prevent them from seeking the help necessary to address the same hindering symptoms. Overall, stigma clearly arose as a barrier to help-seeking in our study, echoing findings in both the general population^34^ and among the broader PSP population.^16^

### Policy Recommendations

Policy considerations for communicators should target 3 levels of support—federal policy, public safety communications leadership, and the individual communicator—to affect their lack of help-seeking behaviors.

At the level of the Canadian federal government, the needs of communicators must underpin policies. There needs to be greater understanding and recognition of the field of public safety communicators so that the public will be neither hostile nor harassing to communicators when calling 911. At present, confusion exists at governmental levels as to the occupational classification of communicators. Guidelines for communications workplaces are neither universal nor clear. For example, in a 2016 report from Oliphant to the Canadian Government, “dispatch officers” were to be part of the first responders and public safety officers' population.^35^ This change has yet to materialize in law. Without this recognition, communicators remain barred from certain privileges awarded in some jurisdictions to first responders (eg, presumptive legislation, Memorial Grant inclusion for families of PSP who die as a result of their duties). Legislative protections against repercussions for those who seek help or disclose mental health needs would start to address the fear some employees hold about job security that prevents help-seeking.^9,10^ Such legislation should include prescribed allowances for time off for mental health help-seeking and recovery to empower communicators to address their mental health struggles. To alleviate fears of financial repercussions and unemployment, legislation should include protections against being dismissed from one's role due to mental health issues.

Moreover, the terminology is ambiguous (what is a public safety communicator verses 911 operator?). Decisions should be made about how to refer to this public safety population in order to recognize them and their needs and to include them in the public safety ecosystem. In addition, it is necessary to distinguish their role and unique needs from other PSP. In the current article, we used the term *public safety communicator* to underpin their ties to public safety, include them as part of the PSP family, and define their role in facilitating communications within public safety responses. An encompassing and universal term to refer to this population can help to streamline policies and increase public recognition of their initiating role in the preservation of life.

The organizational structure of communicator workplaces is regulated at different levels (eg, municipal, private, provincial). Thus, there may be a lack of coordination in services offered, which creates the potential for gaps in delivery, content, and reach of available programs.^36^ A comprehensive census of public safety answering points (PSAPs) introduced within federal policy would further support communicators by allowing those seeking to introduce treatment or conduct research on this underrecognized group to locate PSAPs and to be able to determine the population size—2 unknowns currently in Canada.

The unique environment in which communicators work (ie, never from home, complex workstations, sedentary positions) requires recognition within federal policy, as well as effort from those in public safety communications leadership, to provide proactive support of their physical, social, and mental health. Leadership roles can include direct involvement in public safety communications workplaces. People in such roles include public safety service chiefs, and those in management or supervisory roles. Because poor health and wellbeing can contribute to loss of staff and to increased use of health-related leave, more must be done to maintain the health of this population. Examples of what should be done include ergonomic assessments, exercise opportunities, nutritional consultations, mental health assessments, and team-building activities.

Communicators need training in areas of health and wellbeing, which can help to counter stigma, increase recognition of compromised health, and encourage help-seeking. This training should be effective and evidence informed. It should be evaluated.^16^ In addition, leaders need better understanding of the conditions affecting communicators in each PSAP. Understanding what creates barriers to help-seeking is required to ensure interventions are effective. Open communication between leadership and front-line communicators is necessary to help leaders make informed decisions.

Policy must consider individual factors at the level of the communicators themselves. Help-seeking can be encouraged by reducing or removing stigma from workplace culture. This requires the involvement of the individual communicators in discussions of their wellbeing. Individual communicators affect public safety and public health. They require support to perform their occupational responsibilities. They must be recognized, rather than forgotten, to strengthen their roles in support for the safety and wellness of communities.

### Limitations

The survey question was intentionally broad to allow respondents to explore and report their experiences. As a result, each answer was highly dependent on each participant's interpretation of the question. Moreover, the survey did not allow for follow-up questions or clarifying probes. Future work should employ methods such as interviews to gain richer data. Our survey was administered during the global COVID-19 pandemic which may have affected the experiences of our participants, though our findings notably agree with current understandings of help-seeking in the broader PSP population.

## Conclusions

In our study, we identified diverse factors which affect help-seeking behavior in public safety communicators. Some factors identified by our study are in agreement with findings from prior studies of PSP populations while others were newly identified by this study. As a whole, these findings on public safety communicators allow for a deeper understanding of this under-researched, and often forgotten, PSP group. The revealed themes warrant continued investigation into promoting help-seeking among communicators, creating better workplaces, fostering wellness within communications personnel, and extending such improvements into the communities served through more efficient and well-staffed communications centers. Supportive networks may promote help-seeking behavior. Accommodating work environments may help communicators to gain access to services, without fear of workplace repercussions. These findings highlight the need for the development of policy supportive of the wellbeing of this occupational group, policies that are currently absent within a Canadian context.

## References

[1] Ricciardelli R Groll D Czarnuch S , et al. Behind the frontlines: Exploring the mental health and help-seeking behaviours of public safety personnel who work to support traditional frontline operations. Annu Rev Interdiscip Justice Res 2019; 8: 315.

[2] Adams K Shakespeare-Finch J Armstrong D . An interpretative phenomenological analysis of stress and well-being in emergency medical dispatchers. J Loss Trauma 2015; 20(5): 430–448.

[3] Golding SE Horsfield C Davies A , et al. Exploring the psychological health of emergency dispatch centre operatives: A systematic review and narrative synthesis. PeerJ 2017; 5: e3735. 10.7717/peerj.3735 PMC5649589 29062596

[4] Carleton RN Afifi TO Taillieu T , et al. Exposures to potentially traumatic events among public safety personnel in Canada. Can J Behav Sci Rev Can Sci Comport 2019; 51(1): 37–52.

[5] Carleton RN Afifi TO Turner S , et al. Mental health training, attitudes toward support, and screening positive for mental disorders. Cogn Behav Ther 2020; 49(1): 55–73. 30794073 10.1080/16506073.2019.1575900

[6] Carleton RN Afifi TO Turner S , et al. Mental disorder symptoms among public safety personnel in Canada. Can J Psychiatry 2018; 63(1): 54–64. 28845686 10.1177/0706743717723825 PMC5788123

[7] Government of Canada. Mental Health Disorders in Canada, 2022. *Statistics Canada*, https://www150.statcan.gc.ca/n1/pub/11-627-m/11-627-m2023053-eng.htm (2023, accessed 17 February 2025).

[8] Government of Canada. Mental health care needs, 2018. *Statistics Canada*, https://www150.statcan.gc.ca/n1/pub/82-625-x/2019001/article/00011-eng.htm (2019, accessed 7 December 2022).

[9] Brouwers EPM Joosen MCW van Zelst C , et al. To Disclose or Not to Disclose: A Multi-stakeholder Focus Group Study on Mental Health Issues in the Work Environment. J Occup Rehabil 2020; 30(1): 84–92. 31410722 10.1007/s10926-019-09848-z PMC7031172

[10] Brohan E Henderson C Wheat K , et al. Systematic review of beliefs, behaviours and influencing factors associated with disclosure of a mental health problem in the workplace. BMC Psychiatry 2012; 12, 11. 10.1186/1471-244X-12-11 PMC3298486 22339944

[11] Brouwers EPM . Social stigma is an underestimated contributing factor to unemployment in people with mental illness or mental health issues: Position paper and future directions. BMC Psychol 2020; 8(1): 36. 32317023 10.1186/s40359-020-00399-0 PMC7171845

[12] Burns C Buchanan M . Factors that influence the decision to seek help in a police population. Int J Environ Res Public Health 2020; 17(18): E6891. 10.3390/ijerph17186891 PMC7559930 32967171

[13] Berg AM Hem E Lau B , et al. Help-seeking in the Norwegian police service. J Occup Health 2006; 48(3): 145–153. 16788274 10.1539/joh.48.145

[14] Lane J Le M Martin K , et al. Police attitudes toward seeking professional mental health treatment. J Police Crim Psychol 2022; 37(1): 123–131.

[15] Krakauer RL Stelnicki AM Carleton RN . Examining mental health knowledge, stigma, and service use intentions among public safety personnel. Front Psychol 2020; 11: 949. 32547443 10.3389/fpsyg.2020.00949 PMC7273931

[16] Karaffa KM Koch JM . Stigma, pluralistic ignorance, and attitudes toward seeking mental health services among police officers. Crim Justice Behav 2016; 43(6): 759–777.

[17] Kilpatrick DG Resnick HS Milanak ME , et al. National estimates of exposure to traumatic events and PTSD prevalence using DSM-IV and DSM-5 criteria. J Trauma Stress 2013; 26(5): 537–547. 24151000 10.1002/jts.21848 PMC4096796

[18] Carleton RN Afifi TO Taillieu T , et al. Assessing the relative impact of diverse stressors among public safety personnel. Int J Environ Res Public Health 2020; 17(4): 1234. 32075062 10.3390/ijerph17041234 PMC7068554

[19] Ramey SL Perkhounkova Y Hein M , et al. Evaluation of stress experienced by emergency telecommunications personnel employed in a large metropolitan police department. Workplace Health Saf 2017; 65(7): 287–294. 27941089 10.1177/2165079916667736

[20] Goffman E . Stigma: Notes on the Management of Spoiled Identity. New York, NY: Simon and Schuster, 1963.

[21] Patten SB Williams JVA Lavorato DH , et al. Perceived stigma among recipients of mental health care in the general Canadian population. Can J Psychiatry 2016; 61(8): 480–488. 27310227 10.1177/0706743716639928 PMC4959645

[22] Kronenberg M Osofsky HJ Osofsky JD , et al. First Responder Culture: Implications for Mental Health Professionals Providing Services Following a Natural Disaster. Psychiatr Ann 2008; 38(2): 114–118.

[23] Haugen PT McCrillis AM Smid GE , et al. Mental health stigma and barriers to mental health care for first responders: A systematic review and meta-analysis. J Psychiatr Res 2017; 94: 218–229. 28800529 10.1016/j.jpsychires.2017.08.001

[24] Papazoglou K . Conceptualizing police complex spiral trauma and its applications in the police field. Traumatology 2013; 19(3): 196–209.

[25] McCall HC Landry CA Ogunade A , et al. Why do public safety personnel seek tailored internet-delivered cognitive behavioural therapy? An observational study of treatment-seekers. Int J Environ Res Public Health 2021; 18(22): 11972. 34831728 10.3390/ijerph182211972 PMC8619750

[26] Jones S Agud K McSweeney J . Barriers and facilitators to seeking mental health care among first responders: “removing the darkness”. J Am Psychiatr Nurses Assoc 2020; 26(1): 43–54. 31509058 10.1177/1078390319871997

[27] Dhakal K . NVivo. J Med Libr Assoc JMLA 2022; 110(2): 270–272. 35440911 10.5195/jmla.2022.1271 PMC9014916

[28] Saldaña J . The coding manual for qualitative researchers. 2nd ed. Los Angeles: Sage, 2013.

[29] Mental Health Commission of Canada. The Road To Mental Readiness (R2MR) - One Page Overview, https://mentalhealthcommission.ca/resource/the-road-to-mental-readiness-r2mr-one-page-overview/ (2014, accessed 21 December 2022).

[30] CIPSRT. AMStrength, https://www.cipsrt-icrtsp.ca/en/policy-brief/amstrength (2022, accessed 21 December 2022).

[31] Stanley IH Hom MA Chu C , et al. Perceptions of belongingness and social support attenuate PTSD symptom severity among firefighters: A multistudy investigation. Psychol Serv 2019; 16(4): 543–555. 29595287 10.1037/ser0000240 PMC6163099

[32] Mildenhall J . Occupational stress, paramedic informal coping strategies: A review of the literature. J Paramed Pract 2013; 4(6): 318–328.

[33] Huynh JY Xanthopoulou D Winefield AH . Social support moderates the impact of demands on burnout and organizational connectedness: A two-wave study of volunteer firefighters. APA PsycNET 2013; 18(1): 9–15. 10.1037/a0030804 23276192

[34] Corrigan P . How stigma interferes with mental health care. Am Psychol 2004; 59(7): 614–625. 15491256 10.1037/0003-066X.59.7.614

[35] Oliphant R . *Healthy Minds, Safe Communities: Supporting Our Public Safety Officers Through a National Strategy for Operational Stress Injuries*. 5. The Standing Committee on Public Safety and National Security, 2016. https://www.publicsafety.gc.ca/lbrr/archives/cn37509-eng.pdf.

[36] Public Safety Canada. Supporting Canada’s Public Safety Personnel: An Action Plan on Post-Traumatic Stress Injuries, https://www.publicsafety.gc.ca/cnt/rsrcs/pblctns/2019-ctn-pln-ptsi/index-en.aspx (2019, accessed 18 December 2023).

